# Associations between dietary and supplemental vitamin A intake and melanoma and non‐melanoma skin cancer

**DOI:** 10.1002/ski2.462

**Published:** 2024-10-02

**Authors:** Vaishali Mittal, Jodi Y. So, Shufeng Li, Susan M. Swetter, Eleni Linos, Linda Van Horn, Marian L. Neuhouser, Marcia L. Stefanick, Jean Y. Tang

**Affiliations:** ^1^ Department of Dermatology Stanford University School of Medicine Stanford California USA; ^2^ Department of Urology Stanford University School of Medicine Stanford California USA; ^3^ Dermatology Service Veterans Affairs Palo Alto Health Care System Pala Alto California USA; ^4^ Department of Preventive Medicine Feinberg School of Medicine Northwestern University Chicago Illinois USA; ^5^ University of Washington School of Public Health Seattle Washington USA; ^6^ Fred Hutch Cancer Center Seattle Washington USA; ^7^ Prevention Research Center Stanford University School of Medicine Stanford California USA

## Abstract

**Background:**

Cutaneous melanoma (CM) and non‐melanoma skin cancer (NMSC) are rising in postmenopausal women. Although high doses of oral vitamin A reduce NMSC risk in high‐risk patients, the role of vitamin A in preventing skin cancer in this group remains unexplored.

**Objectives:**

To determine the association between total (dietary and supplemental) vitamin A and risk of CM and NMSC in postmenopausal women.

**Methods:**

This retrospective cohort study included 52 877 White women from the Women's Health Initiative cohort, spanning from 1993 to 2019. Exposures were intake of total vitamin A, retinol and provitamin A carotenoids. Cox proportional hazard models estimated hazard ratios for overall CM incidence, whereas logistic regression determined odds ratios (ORs) for melanoma subtypes and NMSC.

**Results:**

1154 cases of CM and 9085 cases of NMSC were identified over an average follow‐up period of 17.8 years (SD 6.7). No associations were identified between total vitamin A intake and melanoma risk. Higher dietary vitamin A intake was associated with higher risk of NMSC (OR of 3rd vs. 1st tertile of dietary intake = 1.12, 95% confidence interval [CI] [1.06, 1.18]), as was dietary beta‐cryptoxanthin, a provitamin A carotenoid (OR of 3rd vs. 1st tertile of dietary intake = 1.22, 95% CI [1.15, 1.29]); these results were consistent across both age‐ and fully adjusted regression models.

**Conclusions:**

Total vitamin A intake was not associated with lower risk of CM or NMSC. Dietary vitamin A and beta‐cryptoxanthin intake were associated with a slightly higher risk of NMSC in postmenopausal women.

**What is already known about this topic?**

- Incidence of cutaneous melanoma (CM) and non‐melanoma skin cancer (NMSC) is rising among postmenopausal women, and chemoprevention for skin cancer remains an active area of research. High doses of oral vitamin A lower NMSC risk among high‐risk patients in clinical trials. However, the role of dietary and supplemental vitamin A in skin cancer prevention remains unexplored in postmenopausal women despite rising rates in this population.

**What does this study add?**

- We did not find a protective association between dietary and supplemental vitamin A and risk of CM or NMSC. Dietary vitamin A and beta‐cryptoxanthin intake were associated with a small but higher risk for NMSC among postmenopausal women.

## 1 INTRODUCTION

Skin cancer remains the most common form of cancer across the United States,^1^, ^2^ and the incidence of non‐melanoma skin cancer (NMSC), including basal cell carcinoma (BCC), squamous cell carcinoma (SCC) and cutaneous melanoma (CM) in older women has risen markedly in recent years.^3^, ^4^ Sunlight exposure, skin type, personal or family history of skin cancer and history of immunosuppression are known major risk factors for skin cancer.^5^, ^6^, ^7^, ^8^ Ultraviolet (UV) radiation from sunlight is essential for the synthesis of vitamin D3, which is crucial for bone health and immune function.^8^ However, it also has adverse effects on the skin, including the generation of UV‐induced DNA damage, reactive oxygen species and free radicals that promote carcinogenesis.^9^, ^10^, ^11^

Vitamin A refers to a group of compounds, including retinoids, retinol, retinal and retinoic acid among others. Vitamin A derived from animal‐based foods is called retinol. Vitamin A derived from fruits and vegetables is called provitamin A carotenoid and includes beta carotene, alpha carotene and beta cryptoxanthin. After oral consumption, all these compounds undergo conversion to retinal within the human body. Retinal is converted to the most active form of vitamin A in the human body—retinoic acid.^12^ Vitamin A is also thought to potentially reduce skin cancer risk through its mediating effects on cell differentiation, proliferation and apoptosis. Dietary intake of vitamin A has been hypothesised as potentially chemoprotective against skin cancer given the known antioxidant properties.^13^, ^14^

Vitamin A, in its various forms, has emerged as a key intervention to reduce skin cancer risk.

Retinoids are natural and synthetic analogues of retinol and are compounded into systemic and topical agents for the treatment of various skin disorders. Systemic synthetic retinoids include isotretinoin, etretinate and acitretin. Topical synthetic retinoids include tretinoin, adapalene and tazarotene. Systemic retinoids have been shown to be effective chemotherapeutic agents in studies of patients with xeroderma pigmentosum (isotretinoin 2 mg/kg/day)^15^, ^16^ and recipients of organ or bone marrow transplantation (acitretin 25–50 mg/day).^17^, ^18^, ^19^ In addition, patients who do not have these disorders but who are actively developing large numbers of new skin cancers may also benefit from this approach. However, high doses, typically 25–50 mg/day, of retinoids are associated with intolerable side effects including liver dysfunction, hypercholesterolaemia, hypertriglyceridemia, joint and muscle pain, dry mouth, headache and hair loss.^20^ Topical retinoids lack systemic side effects, but daily application of topical retinoids failed to reduce NMSC in a large trial in older men.^21^ Additionally, clinical trials investigating associations between dietary or supplemental vitamin A intake and risk of melanoma or NMSC have yielded no clear relationships,^22^, ^23^, ^24^, ^25^ and to date, there have been no prior investigations correlating both dietary and serologic levels of vitamin A intake with melanoma or NMSC specifically among older women, despite the heightened risk of skin cancer among this at‐risk population. Thus, it is important to examine the role of dietary and supplemental vitamin A in chemoprevention of melanoma and NMSC.

Using data from a longitudinal, prospective, observational study, we sought to explore associations between dietary, supplemental and total (dietary + supplemental) intake and serologic levels of vitamin A and related compounds, and incidence of melanoma and NMSC in a large cohort of postmenopausal women enroled in the Women's Health Initiative (WHI).

## 2 METHODS

### 2.1 Data source

To investigate associations between vitamin A and skin cancer in postmenopausal women, we used data from the observational arm (OS) of the WHI (NCT00000611), which is a national, prospective cohort study of risk factors and modifiers, and biological disease markers in postmenopausal women.^26^ The study design of the OS has been previously and extensively detailed^27^; in brief, from 1993 to 1998, the OS enroled a geographically diverse cohort of 93 676 postmenopausal women at over 40 clinical centres across the United States.

Because sun exposure is a significant known risk factor for skin cancer, we used data from the OS, rather than the full WHI cohort, as detailed sun exposure history was only captured for participants in the OS. This study was approved by the Institutional Review Board for each individual site, and all participants provided written informed consent.

We analysed the survey data captured at baseline including demographics, daily dietary vitamin A intake estimated by Food Frequency Questionnaires (FFQ), daily supplemental vitamin A intake, medical history, tendency to sunburn/tan and childhood and current sun exposure in minutes.

Analyses were restricted to White participants because non‐White individuals have lower risk of skin cancer.^3^ We additionally excluded participants with missing data on diet or supplement use at baseline, with unreliable dietary data (e.g. reported caloric intake of <600 NMSCal/day or >5000 NMSCal/day) or with missing data for any of the covariates of interest, including prior cancer history.

### 2.2 Assessment of melanoma and NMSC cases

The primary outcomes of interest were incidence of melanoma and NMSC through the end of follow‐up on 1 March 2019. The WHI does not distinguish between SCC and BCC so we combined these into NMSC and analysed both in situ and invasive SCC. Cases of melanoma were reported by participants and subsequently confirmed and staged by trained, centralised adjudicators through detailed review of medical records and histopathologic reports. New diagnoses of NMSC were self‐reported at annual visits but were not centrally adjudicated.

### 2.3 Assessment of exposures

We examined different types of vitamin A exposure, including both dietary sources and supplements. We defined dietary intake as the consumption derived from food. Total vitamin A intake was evaluated at baseline through two sources: (1) dietary intake and (2) supplement intake. Additionally, we assessed dietary retinol and total retinol. Furthermore, we analysed provitamin A forms obtained from fruits and vegetables, such as dietary alpha‐carotene, total and dietary beta‐carotene and dietary beta‐cryptoxanthin. All these forms are converted into retinoic acid, which is the active form of vitamin A.

Daily dietary intake of individual vitamins and nutrients were assessed through the validated FFQ (Form 60),^28^ which calculated daily intake of individual nutrients based on participants' report of 122 foods and food groups over the prior 3 months. It is worth noting that this 3‐month period was not a requirement for inclusion. The exposure to vitamin intake was calculated as the average value over the first three visits of the study, including the baseline visit at year 0, the visit at year 1 and the visit at year 2. Participants were also instructed to bring bottles of all vitamin and nutrient supplements used at the baseline visit, where clinic staff reviewed and transcribed current daily supplement use and doses on certain micronutrients that included carotenoids and vitamin A (Form 60). Lastly, fasting blood samples were drawn at baseline visits and subsequently analysed for individual serum levels of analytes in 1% of randomly selected OS participants (*n* = 1062), using high‐performance liquid chromatography with UV detection.

### 2.4 Covariates

Detailed participant information including demographics, medical history, smoking status, current and past sun exposure, sun exposure habits (e.g. sunscreen use and average sunscreen sun protection factor [SPF]), tendency to burn or tan and solar irradiation of the respondent's geographic location^29^ (in Langleys; 1 Langley = 1 g‐cal/cm^2^]) were captured at annual clinical visits through clinical examinations, interviews or surveys. Although we could not adjust for physical activity, which might serve as an indirect measure of outdoor exposure, we carefully controlled for sun exposure by considering the time spent outdoors during childhood, teenage years, thirties and the current year of the study. Sun exposure history and habits were assessed through a questionnaire (Form 144) administered during year 4 and included key questions regarding sun exposure including skin reaction to the sun after 45–60 min of exposure, daily time spent outdoors in the summer during childhood and at present, daily sunscreen use and SPF of daily sunscreen (if applicable).

### 2.5 Statistical analysis

Vitamin intake and serum levels were stratified by tertile, with the lowest tertile as the reference.

Vitamin A‐related nutrients investigated in this study were as follows: dietary and supplemental vitamin A intake (mcg/day), dietary and supplemental retinol intake (mcg/day), dietary and supplemental beta‐carotene intake (mcg/day), dietary intake of individual alpha‐carotene (mcg/day) and dietary intake of beta‐cryptoxanthin (mcg/day). Vitamin A was calculated as the sum of retinol and provitamin A carotenoids, adjusted by their vitamin A activity and bioavailability in retinol activity equivalents.^29^ ^,^ ^30^ The recommended daily allowance (RAE) for vitamin A is 900 mcg for adults and children aged 4 years and older, where 1 mcg RAE = 1 mcg retinol, 2 mcg beta‐carotene from supplements, 12 mcg beta‐carotene from foods, 24 mcg alpha‐carotene or 24 mcg beta‐cryptoxanthin. Total daily intake was the sum of daily intake via diet and supplements. Serum levels of individual compounds were also analysed: retinol (μ/mL), alpha‐carotene (μ/mL), beta‐carotene (μ/mL) and beta‐cryptoxanthin (μ/mL).

Participant characteristics were analysed by counts and percentages and compared across tertiles by chi‐square tests. To examine relationships between vitamin intake (by tertile) and risk of melanoma, Cox proportional hazard models were developed to estimate hazard ratios (HRs) associated with incidence of melanoma of any subtype (e.g. both in situ and invasive disease). Cox regressions could only be performed for incidence of melanoma as only melanoma had time‐to‐diagnosis recorded. Logistic regressions were then used to estimate odds ratios (OR) associated with either melanoma in situ or invasive melanoma among incident cases. To assess the overall risk of NMSC, logistic regressions were developed to estimate OR of NMSC. All regressions adjusted for either age alone, or for all covariates including socio‐demographics, medical history and sun exposure. As a sensitivity analysis, age‐ and fully adjusted binomial logistic regression models were also used to assess the associations between serum vitamin levels and odds of NMSC among participants with available serologic data; serum sensitivity analyses were not performed for melanoma due to the very small sample of participants who both had serologic data available and developed melanoma during the study period. Statistical analyses were completed in Stata 16.1 and SAS 9.4 (SAS Institute Inc.). All statistical tests were two‐sided, and *p* < 0.05 were considered significant.

## 3 RESULTS

The final cohort comprised 52 877 White women with mean 17.8 years of follow‐up (SD 6.7 years, range 3.2–26.4 years) (Table 1). Women in the highest tertile of vitamin A intake were more likely to be older and highly educated, to have a prior history of NMSC or a non‐skin cancer, and to use sunscreen with higher SPF; those in the highest tertile of vitamin A intake were less likely to be obese or have extended sun exposure during childhood or at present.

**TABLE 1:** Baseline characteristics of participants by total vitamin A intake (dietary and supplements) in the Women's Health Initiative.

Characteristic	Total, *N* (%)	Total vitamin A intake^a^			
		1st tertile	2nd tertile	3rd tertile	*p*‐value^b^ ^,^ ^c^
		[27–88 815 mcg]	[888–5162 mcg]	[5162–80 505 mcg]	
		*N* (%)	*N* (%)	*N* (%)	
New diagnosis of melanoma	1154 (2.2)	360 (2.0)	397 (2.3)	397 (2.3)	
Tumour behaviour at diagnosis					0.93
In situ disease	545 (47.2)	172 (1.0)	183 (1.0)	190 (1.1)	
Invasive disease	595 (51.6)	184 (1.0)	210 (1.2)	201 (1.1)	
Unknown	14 (1.2)	4 (0)	4 (0)	6 (0)	
New diagnosis of keratinocyte cancer (NMSC)	9832 (18.6)	3087 (17.5)	3355 (19.0)	3390 (19.2)	**<0.01**
Age					**<0.01**
50–59	16 989 (32.1)	6160 (35.0)	5511 (31.3)	5318 (30.2)	
60–69	23 773 (45.0)	7749 (44.0)	7978 (45.3)	8046 (45.6)	
70+	12 115 (22.9)	3716 (21.1)	4137 (23.5)	4262 (24.2)	
Education					**<0.01**
None to some high school	1430 (2.7)	628 (3.6)	446 (2.5)	356 (2.0)	
High school diploma or GED	8387 (15.9)	3300 (18.7)	2720 (15.4)	2367 (13.4)	
Some college, college degree or higher	43 060 (81.4)	13 697 (77.7)	14 460 (82.0)	14 903 (84.6)	
Income					**0.02**
<$20 000–$49 999	29 369 (55.5)	9921 (56.3)	9826 (55.7)	9622 (54.6)	
$50 000–$99 999	16 895 (32.0)	5520 (31.3)	5597 (31.8)	5778 (32.8)	
≥$100 000	6613 (12.5)	2184 (12.4)	2203 (12.5)	2226 (12.6)	
Body mass index (kg/m^2^)					**<0.01**
<18.5 (underweight)	542 (1.0)	163 (0.9)	172 (1.0)	207 (1.2)	
18.5–24.9 (normal weight)	22 218 (42.0)	7130 (40.5)	7343 (41.7)	7745 (43.9)	
≥25 (overweight or obese)	30 117 (57.0)	10 332 (58.6)	10 111 (57.4)	9674 (54.9)	
Smoking history					**<0.01**
Never	26 448 (50.0)	8657 (49.1)	9022 (51.2)	8769 (49.8)	
Past	23 717 (44.9)	7804 (44.3)	7781 (44.1)	8132 (46.1)	
Current	2712 (5.1)	1164 (6.6)	823 (4.7)	725 (4.1)	
Prior history of melanoma	5005 (9.5)	1475 (8.4)	1666 (9.5)	1864 (10.6)	0.39
Prior history of NMSC	6084 (11.5)	1885 (10.7)	2022 (11.5)	2177 (12.4)	**<0.01**
Prior history of non‐skin cancer	36 252 (68.6)	12 033 (68.3)	12 080 (68.5)	12 139 (68.9)	**<0.01**
Family history of cancer	46 594 (88.1)	15 301 (86.8)	15 499 (87.9)	15 794 (89.6)	0.48
Had last medical visit within 1 year	5005 (9.5)	1475 (8.4)	1666 (9.5)	1864 (10.6)	**<0.01**
Regional solar irradiation (in Langleys)					**<0.01**
300–325	17 115 (32.4)	5868 (33.3)	5957 (33.8)	5290 (30.0)	
350	11 021 (20.8)	3469 (19.7)	3704 (21.0)	3848 (21.8)	
375–380	5686 (10.8)	2089 (11.9)	1825 (10.4)	1772 (10.1)	
400–430	8707 (16.5)	2899 (16.4)	2748 (15.6)	3060 (17.4)	
475–500	10 348 (19.6)	3300 (18.7)	3392 (19.2)	3656 (20.7)	
Skin reaction to sun					**0.01**
Does not burn	18 878 (35.7)	6495 (36.9)	6235 (35.4)	6131 (34.8)	
Burns, then tans	13 596 (25.7)	4435 (25.2)	4550 (25.9)	4588 (26.1)	
Burns, then tans a minimal amount	14 440 (27.3)	4664 (26.5)	4827 (27.4)	4926 (28.0)	
Burns but does not tan	5963 (11.3)	2007 (11.4)	1989 (11.3)	1956 (11.1)	
Average daily sun exposure in summer during childhood					0.25
<30 min	1155 (2.2)	412 (2.3)	392 (2.2)	351 (2.0)	
30 min–2 h	13 703 (25.9)	4554 (25.8)	4549 (25.8)	4600 (26.1)	
>2 h	38 019 (71.9)	12 659 (71.8)	12 685 (72)	12 675 (71.9)	
Average daily sun exposure in summer now					**<0.01**
<30 min	16 088 (30.4)	5311 (30.1)	5341 (30.3)	5436 (30.8)	
30 min–2 h	26 618 (50.3)	8746 (49.6)	8933 (50.7)	8939 (50.7)	
>2 h	10 171 (19.2)	3568 (20.2)	3352 (19.0)	3251 (18.4)	
Sunscreen SPF					**<0.01**
None	24 586 (46.5)	9029 (51.2)	8183 (46.4)	7374 (41.8)	
SPF 2–14	2609 (4.9)	894 (5.1)	878 (5.0)	837 (4.7)	
SPF 15–25	16 300 (30.8)	4935 (28.0)	5414 (30.7)	5951 (33.8)	
SPF 25+	9382 (17.7)	2767 (15.7)	3151 (17.9)	3464 (19.7)	

Abbreviation: CI, confidence interval; GED, general educational development.

^a^ Total intake includes intake via diet and supplements. Dietary intake is defined as consumption derived from food. All units are micrograms.

^b^ Bolded values indicate two‐sided statistical significance at the *p* < 0.05 level.

^c^ Chi‐square test was used.

1154 adjudicated cases of melanoma occurred during the study period; of this, 51.6% (*n* = 595/1154) of melanoma cases were in situ, 47.2% (545/1154) were diagnosed with invasive melanoma and 1.2% (14/1154) had unspecified tumour behaviour at diagnosis. In age‐ and fully adjusted analyses, there were no significant differences in risk of a new melanoma diagnosis or risk of invasive melanoma associated with total or dietary vitamin A, or any retinol or provitamin A carotenoids (Table 2).

**TABLE 2:** Age‐ and multivariate‐adjusted associations between vitamin intake and melanoma.^a^

Vitamin intake^b^	No. of cases	New melanoma diagnosis			
		Age‐adjusted HR^c^ ^,^ ^d^ (95% CI)	*p*‐value	Fully adjusted HR^d^ ^,^ ^e^ (95% CI)	*p*‐value
Total vitamin A					
1st tertile [27–888] (*reference*)	360	‐		‐	
2nd tertile [888–5162]	397	1.11 (0.96, 1.28)	0.16	1.05 (0.91, 1.21)	0.49
3rd tertile [5162–80505]	397	1.11 (0.96, 1.27)	0.176	1.01 (0.87, 1.17)	0.90
Dietary vitamin A					
1st tertile [27–562] (*ref*)	383	‐		‐	
2nd tertile [562–834]	387	1.00 (0.87, 1.15)	0.97	0.95 (0.83, 1.10)	0.50
3rd tertile [834–11320]	384	1.01 (0.88, 1.16)	0.90	0.96 (0.83, 1.11)	0.58
Total retinol					
1st tertile [6–474] (*ref*)	381	‐		‐	
2nd tertile [474–1124]	378	1.00 (0.87, 1.16)	0.97	0.98 (0.85, 1.13)	0.74
3rd tertile [1124–33420]	395	1.05 (0.91, 1.21)	0.51	1.00 (0.87, 1.16)	0.96
Dietary retinol					
1st tertile [1–286] (*ref*)	402	‐		‐	
2nd tertile [286–476]	376	0.94 (0.82, 1.08)	0.38	0.94 (0.81, 1.08)	0.36
3rd tertile [476–10849]	376	0.96 (0.83, 1.10)	0.53	0.97 (0.84, 1.12)	0.70
Dietary alpha‐carotene					
1st tertile [0–18] (*ref*)	349	‐		‐	
2nd tertile [18–34]	401	1.12 (0.97, 1.30)	0.11	1.05 (0.91, 1.21)	0.50
3rd tertile [34–424]	404	1.12 (0.97, 1.30)	0.11	1.01 (0.87, 1.17)	0.93
Total beta‐carotene					
1st tertile [5–302] (*ref*)	364	‐		‐	
2nd tertile [302–2420]	387	1.05 (0.91, 1.21)	0.49	0.99 (0.85, 1.14)	0.84
3rd tertile [2420–39436]	403	1.10 (0.96, 1.27)	0.18	0.98 (0.85, 1.14)	0.83
Dietary beta‐carotene					
1st tertile [5–182] (*ref*)	379	‐		‐	
2nd tertile [182–313]	384	1.00 (0.86, 1.15)	0.96	0.92 (0.79, 1.06)	0.22
3rd tertile [313–4760]	391	1.02 (0.89, 1.17)	0.79	0.90 (0.78, 1.04)	0.14
Dietary beta‐cryptoxanthin					
1st tertile [0–4] (*ref*)	354	‐		‐	
2nd tertile [4–7]	396	1.10 (0.96, 1.27)	0.18	1.03 (0.89, 1.19)	0.69
3rd tertile [7–42]	404	1.12 (0.97, 1.29)	0.12	1.01 (0.88, 1.17)	0.88

Abbreviation: CI, confidence interval.

^a^ Bolded values indicate two‐sided statistical significance at the *p* < 0.05 level.

^b^ Total intake includes intake via diet and supplements. Dietary intake is defined as consumption derived from food. All units are micrograms.

^c^ Adjusted for age.

^d^ HR, hazard ratio, assessed by Cox proportional hazard models.

^e^ Adjusted for age, education, income, body mass index, smoking history, prior history of melanoma, prior history of non‐melanoma skin cancer, prior history of non‐skin cancer, family history of cancer, had last medical visit within 1 year, solar irradiation of the participant's geographic location of residence, skin reaction to sun exposure, average daily sun exposure in summer during childhood, average daily sun exposure in summer now and sunscreen use and sun protection factor (SPF).

9085 cases of NMSC were reported by participants. In age‐adjusted analyses, higher intake of total vitamin A (OR of 3rd tertile = 1.10, 95% confidence interval [CI] [1.04, 1.16]), dietary vitamin A (OR of 3rd tertile = 1.12, 95% CI [1.06, 1.18]), total retinol (OR of 3rd tertile = 1.07, 95% CI [1.01, 1.13]), dietary alpha‐carotene (OR of 3rd tertile = 1.15, 95% CI [1.09, 1.22]), total beta‐carotene (OR of 3rd tertile = 1.13, 95% CI [1.07, 1.20]), dietary beta‐carotene (OR of 3rd tertile = 1.18, 95% CI [1.12, 1.25]) and dietary beta‐cryptoxanthin (OR of 3rd tertile = 1.22, 95% CI [1.15, 1.29]) were associated with increased odds of developing NMSC (Table 3). After adjusting for all covariates, risk of NMSC was positively correlated with greater intake of dietary vitamin A (OR of 3rd tertile = 1.07, 95% CI [1.01, 1.13]), dietary retinol (OR of 3rd tertile = 1.07, 95% CI [1.02, 1.14]), dietary beta‐carotene (OR of 2nd tertile = 1.06, 95% CI [1.00, 1.13]) and dietary beta‐cryptoxanthin (OR of 3rd tertile = 1.22, 95% CI [1.15, 1.29]).

**TABLE 3:** Age‐ and multivariate‐adjusted associations between vitamin intake and NMSC.^a^

Vitamin intake^b^	No. of cases	Age‐adjusted OR^c^ ^,^ ^d^ (95% CI)	*p*‐value	Fully adjusted OR^c^ ^,^ ^e^ (95% CI)	*p*‐value
Total vitamin A					
1st tertile [27–888] (*reference*)	3087	‐		‐	
2nd tertile [888–5162]	3355	**1.09 (1.03, 1.15)**	<0.01	1.03 (0.98, 1.09)	0.26
3rd tertile [5162–80505]	3390	**1.10 (1.04, 1.16)**	<0.01	0.98 (0.93, 1.04)	0.55
Dietary vitamin A					
1st tertile [27–562] (*ref*)	3042	‐		‐	
2nd tertile [562–834]	3381	**1.12 (1.06, 1.18)**	<0.01	**1.08 (1.02, 1.14)**	0.01
3rd tertile [834–11320]	3409	**1.12 (1.06, 1.18)**	<0.01	**1.07 (1.01, 1.13)**	0.02
Total retinol					
1st tertile [6–474] (*ref*)	3134	‐		‐	
2nd tertile [474–1124]	3344	**1.07 (1.01, 1.13)**	0.01	1.05 (0.99, 1.11)	0.09
3rd tertile [1124–33420]	3354	**1.07 (1.01, 1.13)**	0.01	1.02 (0.96, 1.07)	0.61
Dietary retinol					
1st tertile [1–286] (*ref*)	3175	‐		‐	
2nd tertile [286–476]	3323	1.04 (0.99, 1.10)	0.13	1.04 (0.99, 1.10)	0.15
3rd tertile [476–10849]	3334	1.04 (0.99, 1.10)	0.13	**1.07 (1.02, 1.14)**	0.01
Dietary alpha‐carotene					
1st tertile [0–18] (*ref*)	3030	‐		‐	
2nd tertile [18–34]	3336	**1.11 (1.05, 1.17)**	<0.01	1.05 (0.99, 1.11)	0.08
3rd tertile [34–424]	3466	**1.15 (1.09, 1.22)**	<0.01	1.04 (0.98, 1.10)	0.19
Total beta‐carotene					
1st tertile [5–302] (*ref*)	3034	‐		‐	
2nd tertile [302–2420]	3377	**1.13 (1.07, 1.19)**	<0.01	1.04 (0.98, 1.10)	0.17
3rd tertile [2420–39436]	3421	**1.13 (1.07, 1.20)**	<0.01	0.99 (0.94, 1.05)	0.80
Dietary beta‐carotene					
1st tertile [5–182] (*ref*)	2973	‐		‐	
2nd tertile [182–313]	3371	**1.14 (1.08, 1.21)**	<0.01	**1.06 (1.00, 1.13)**	0.04
3rd tertile [313–4760]	3488	**1.18 (1.12, 1.25)**	<0.01	1.04 (0.98, 1.10)	0.16
Dietary beta‐cryptoxanthin					
1st tertile [0–4] (*ref*)	2912	‐		‐	
2nd tertile [4–7]	3427	**1.20 (1.13, 1.26)**	<0.01	**1.14 (1.07, 1.20)**	<0.01
3rd tertile [7–42]	3493	**1.22 (1.15, 1.29)**	<0.01	**1.11 (1.05, 1.18)**	<0.01

Abbreviation: CI, confidence interval.

^a^ Bolded values indicate two‐sided statistical significance at the *p* < 0.05 level.

^b^ Total intake includes intake via diet and supplements. Dietary intake is defined as consumption derived from food. All units are micrograms.

^c^ OR, odds ratio, assessed by logistic regression models.

^d^ Adjusted for age.

^e^ Adjusted for age, education, income, body mass index, smoking history, prior history of melanoma, prior history of non‐melanoma skin cancer, prior history of non‐skin cancer, family history of cancer, had last medical visit within 1 year, solar irradiation of the participant's geographic location of residence, skin reaction to sun exposure, average daily sun exposure in summer during childhood, average daily sun exposure in summer now and sunscreen use and sun protection factor (SPF).

Serologic analysis of individual analytes was performed for only a small subset of 504 women. Of this, 93 participants reported a diagnosis of NMSC during the study period (Table 4). Women with high serum levels of beta‐carotene had 2.7‐fold greater odds of a diagnosis of NMSC (95% CI [1.33, 5.48]); no other individual serum analytes were associated with increased or decreased odds of NMSC.

**TABLE 4:** Age‐ and multivariate‐adjusted associations between serum vitamin levels and non‐melanoma skin cancer.^a^

Serum vitamin levels	No. of cases	Age‐adjusted OR^b^ ^,^ ^c^ (95% CI)	*p*‐value	Fully adjusted OR^b^ ^,^ ^d^ (95% CI)	*p*‐value
Retinol					
1st tertile (0.2–0.5) (*ref*)	35	‐		‐	
2nd tertile (0.5–0.6)	27	0.74 (0.42, 1.29)	0.44	0.61 (0.32, 1.16)	0.13
3rd tertile (0.6–1.3)	31	0.81 (0.47, 1.39)	0.80	0.72 (0.39, 1.35)	0.31
Alpha‐carotene					
1st tertile (0–0.1) (*ref*)	30	‐		‐	
2nd tertile (0.06–0.12)	30	1.04 (0.59, 1.83)	0.95	1.27 (0.67, 2.41)	0.47
3rd tertile (0.12–1.15)	33	1.11 (0.64, 1.93)	0.72	1.49 (0.77, 2.88)	0.23
Beta‐carotene					
1st tertile (0–0.2) (*ref*)	24	‐		‐	
2nd tertile (0.2–04)	33	1.48 (0.83, 2.64)	0.50	1.88 (0.96, 3.69)	0.07
3rd tertile (0.4–2.4)	36	1.57 (0.89, 2.79)	0.29	**2.70 (1.33, 5.48)**	**<0.01**
Beta‐cryptoxanthin					
1st tertile (0–0.1) (*ref*)	29	‐		‐	
2nd tertile (0.1–0.11)	34	1.11 (0.63, 1.93)	0.66	1.49 (0.78, 2.83)	0.23
3rd tertile (0.11–0.6)	30	0.99 (0.56, 1.74)	0.80	1.42 (0.72, 2.82)	0.31

Abbreviation: CI, confidence interval.

^a^ Bolded values indicate two‐sided statistical significance at the *p* < 0.05 level.

^b^ OR, odds ratio, assessed by logistic regression models.

^c^ Adjusted for age.

^d^ Adjusted for age, education, income, body mass index, smoking history, prior history of melanoma, prior history of non‐melanoma skin cancer, prior history of non‐skin cancer, family history of cancer, had last medical visit within 1 year, solar irradiation of the participant's geographic location of residence, skin reaction to sun exposure, average daily sun exposure in summer during childhood, average daily sun exposure in summer now and sunscreen use and sun protection factor (SPF).

## 4 DISCUSSION

In this large prospective cohort study, we examined associations between vitamin A and incidence of CM and NMSC, including BCC and SCC, among postmenopausal women enroled in the WHI observational study.

We found no significant associations between vitamin A intake and CM development. Overall, the lack of observed protective associations between vitamin A intake and melanoma is consistent with prior prospective studies which have yielded mixed results, including in studies which examined skin cancer risk in both pre‐ and post‐menopausal‐aged women.^31^, ^32^, ^33^, ^34^

We found that increased intake of dietary and supplemental vitamin A and beta‐cryptoxanthin, a provitamin A carotenoid, was associated with a small but increased risk for NMSC in postmenopausal women, even after controlling for possible confounders including respondent age, sun exposure history and prior skin cancer history. However, this could be spurious due to several factors such as the absence of physician‐adjudicated NMSC cases, analysing BCC and SCC cases together and the minimal intake levels of beta‐cryptoxanthin. If there is a true association between the intake of dietary and supplemental vitamin A and beta‐cryptoxanthin and increased NMSC, it could be due to several factors. Excessive intake of certain antioxidants, such as beta‐carotene (a provitamin A), can sometimes act as pro‐oxidants at high concentrations, potentially leading to oxidative stress rather than protection. This has been observed in some studies, particularly with high‐dose beta‐carotene supplements, which can paradoxically increase cancer risk by promoting oxidative damage. Additionally, a high intake of beta‐carotene without adequate levels of other essential nutrients might not provide the expected protective effects and could contribute to increased cancer risk.

Our finding is consistent with prior work which have found similar positive associations between beta‐cryptoxanthin and increased NMSC risk among both women aged 30–55.^35^ We conclude that higher total vitamin A levels do not appear to correspond with lower NMSC risk.

Our findings are in contrast with a recent 2019 epidemiologic study which showed that higher total vitamin A and beta‐cryptoxanthin intake may be protective against cutaneous SCC, although this study included male and female study participants, and did not report sex‐stratified results.^23^ This may due to sex differences in NMSC risk factors and biology, including sex‐related differences in cutaneous antioxidant capacity, or UV‐induced inflammatory changes, oxidative DNA damage and tumour development.^34^, ^35^, ^36^ It may also be due to potential interactions between vitamin A intake and other dietary factors that could influence NMSC risk, such as the balance between various dietary antioxidants and prooxidants.

Although systemic retinoids have demonstrated chemoprotective effects, dietary and supplemental retinoids may not offer the same benefits due to their lower bioavailability and efficacy. The body must convert provitamin A into active retinoids, but this conversion is not always efficient. Individual metabolic variations can result in inadequate levels of active retinoic acid, which may fail to protect against skin cancer. Additionally, dietary and supplemental vitamin A lack the potent, targeted effects on gene expression that systemic retinoids achieve through retinoic acid receptors (RARs and RXRs), which significantly influence cell differentiation, proliferation and apoptosis.

While our results are not based on a randomized controlled trial, our study cohort of older women appears to be the first to demonstrate an association between increased vitamin A intake and slightly higher risk of NMSC and shows no protective effect of dietary or supplemental vitamin A on CM or NMSC risk. Further investigation is needed to clarify possible distinct NMSC risk profiles associated with vitamin A and provitamin A carotenoids and to understand both sex and age differences in skin cancer development and chemoprevention strategies.

### 4.1 Strengths and limitations

The study's strengths include the extensive length of follow‐up, use of detailed data on participant socio‐demographic characteristics, medical and family history, validated dietary intake and longitudinal use and dosing of supplements captured by trained interviewers, sun exposure history, adjudicated clinical outcomes and serologic analyte data. However, there are also some limitations. First, some data were self‐reported by participants, which may be subject to inaccuracy or recall bias. Second, data on diet, nutrition and supplement use assessed over the study period were exclusively based on the period of observation within the WHI and may not be representative of participants' lifetime habits. Third, while the number of adjudicated melanoma cases available in preliminary analyses is larger than that of some existing studies evaluating associations between micronutrients and melanoma, the study may not have sufficient power‐specific sub‐analyses, including when comparing in situ and invasive disease. NMSC cases were also not centrally adjudicated and may be subject to recall bias. Lastly, tumour‐specific characteristics were not assessed as the WHI does not capture pathologic, histologic or genetic data for melanoma or NMSC.

## 5 CONCLUSION

In this study, we investigated associations between vitamin A and NMSC and CM and found no protective associations in a large cohort of postmenopausal women; however, dietary vitamin A and beta‐cryptoxanthin, a provitamin A carotenoid, were associated with a slightly increased risk of NMSC. However, this could be a spurious finding and further investigation is necessary to understand the association between vitamin A intake and risk of skin cancer in postmenopausal women.

## CONFLICT OF INTEREST STATEMENT

The authors state no conflicts of interest.

## AUTHOR CONTRIBUTIONS

**Vaishali Mittal**: Data curation (equal); formal analysis (equal); methodology (equal); visualisation (lead); writing—original draft (lead); writing—review and editing (lead). **Jodi Y. So**: Conceptualisation (equal); data curation (equal); methodology (equal). **Shufeng Li**: Formal analysis (supporting). **Susan M. Swetter**: Methodology (supporting); writing—review and editing (supporting). **Eleni Linos**: Writing—review and editing (supporting). **Linda Van Horn**: Methodology (supporting); writing—review and editing (supporting). **Marian L. Neuhouser**: Methodology (supporting); writing—review and editing (supporting). **Marcia L. Stefanick**: Methodology (supporting); supervision (lead); writing—review and editing (supporting). **Jean Y. Tang**: Formal analysis (supporting); methodology (supporting); supervision (lead); writing—review and editing (supporting).

## ETHICS STATEMENT

The WHI project was reviewed and approved by the Fred Hutchinson Cancer Research Centre (Fred Hutch) IRB in accordance with the US Department of Health and Human Services regulations at 45 CFR 46 (approval number: IR# 3467‐EXT). Participants provided written informed consent to participate. Additional consent to review medical records was obtained through signed written consent. Fred Hutch has an approved FWA on file with the Office for Human Research Protections under assurance number 0001920.

## PATIENT CONSENT

Written patient consent for publication was obtained.

## Data Availability

The data underlying this article will be shared on reasonable request to the corresponding author.
